# Molecular Characterization of Community Acquired *Staphylococcus aureus* Bacteremia in Young Children in Southern Mozambique, 2001–2009

**DOI:** 10.3389/fmicb.2017.00730

**Published:** 2017-05-04

**Authors:** Delfino Vubil, Marcelino Garrine, Ulla Ruffing, Sozinho Acácio, Betuel Sigaúque, Pedro L. Alonso, Lutz von Müller, Mathias Herrmann, Inácio Mandomando

**Affiliations:** ^1^Centro de Investigação em Saúde de Manhiça (CISM)Maputo, Mozambique; ^2^Institute of Medical Microbiology and Hygiene, University of SaarlandHomburg, Germany; ^3^Instituto Nacional de Saúde (INS), Ministério da SaúdeMaputo, Mozambique; ^4^Barcelona Institute of Global HealthBarcelona, Spain

**Keywords:** *Staphyloccoccus aureus*, bacteremia, molecular characterization, virulence genes, PVL

## Abstract

**Background:** The emergence of community-acquired *Staphylococcus aureus* infections is increasingly recognized as life threating problem worldwide. In Manhiça district, southern Mozambique, *S. aureus* is the leading cause of community-acquired bacteremia in neonates.

**Methods:** Eighty-four *S. aureus* isolates from children less than 5 years admitted to Manhiça District Hospital from 2001 to 2009 were randomly selected and genetically characterized by DNA microarray and *spa* typing. Antimicrobial susceptibility was determined by VITEK 2.

**Results:** Thirty-eight different *spa* types and 14 clonal complexes (CC) were identified. *Spa*-type t084 (*n* = 10; 12%) was the most predominant while CC8 (*n* = 18; 21%) and CC15 (*n* = 14; 16%) were the most frequent CCs. Mortality tended to be higher among children infected with CC45 (33.3%, 1/3) and CC8 (27.8%, 5/18). The majority of isolates possessed the accessory gene regulator I (45%) and belonged to either capsule type 8 (52%) or 5 (47%). Panton valentine leukocidin (PVL) encoding genes were detected in 30%. Antibiotic resistance was high for penicillin (89%), tetracycline (59%) and Trimethoprim Sulfamethoxazole (36%) while MRSA was uncommon (8%).

**Conclusions:** Although MRSA were uncommon, we found high genetic diversity of methicillin susceptible *S. aureus* causing bacteremia in Mozambican children, associated with high resistance to the most available antibiotics in this community. Some CCs are likely to be more lethal indicating the need for prompt recognition and appropriate treatment.

## Introduction

*Staphylococcus aureus* is an important cause of human infections ranging from skin abscesses to life threatening conditions such as bacteremia and pneumonia. This pathogen is able to cause hospital as well as community acquired infections (Chen and Huang, 2014). These infections are often associated with high rates of antibiotic resistance representing a serious challenge for patient management. Of particular importance, methicillin resistant *S. aureus* (MRSA) has emerged as a notorious etiologic agent for a wide range of infections worldwide (Shittu et al., 2011).

The ability of *S. aureus* to cause multiple infections has been associated with the expression of myriads of different toxins, virulence factors, cell wall adhesion proteins such as MSCRAMMs (Microbial Surface Components Recognizing Adhesive Matrix Molecules) and other proteins involved in immune evasion (Shambat et al., 2012). MSCRAMMs are among the factors of interest, as they are known to have the capacity to bind extracellular matrix proteins such as collagen, fibrinogen and fibronectin, all potentially important for the ability of *S. aureus* invasion. In addition, different *S. aureus* may have different constellations of MSCRAMMs and so may be predisposed to cause certain types of infections (Gordon and Lowy, 2008). Toxins are another group of critical virulence factors, among them the Panton–Valentine Leukocidin (PVL) is of particular importance. PVL is a bi-component (*lukS-PV* and *lukF-PV*) pore-forming cytotoxin that has been shown to target polymorphonuclear cells, monocytes and macrophages in humans and rabbits (Boyle-Vavra and Daum, 2007) and has been associated with a highly aggressive and often fatal form of community acquired infections (Stryjewski and Chambers, 2008). Furthermore, capsular polysaccharides and regulators such as the accessory gene regulatory (*agr*) may also play a role on *S. aureus* pathogenicity. Capsular polysaccharide or capsule is a cell wall bacterial component which protects bacterium from phagocytic and enhances microbial virulence (Verdier et al., 2007) while the *agr* locus is a quorum sensing system, essential for the global regulation of *S. aureus* virulence factors and other accessory gene functions (Chong et al., 2013). Due to polymorphisms in the *agr* locus, the *S. aureus* isolates are assigned to *agr* groups I to IV (Rasmussen et al., 2013).

While *S. aureus* infections from developed countries have been extensively studied concerning their virulence patterns and clonal relatedness, the corresponding data from Africa is limited. Available data have shown differences on clonal structure and virulence patterns of African isolates (Schaumburg et al., 2011), often associated with high mortality compared to those from industrialized countries (Shittu et al., 2011).

In Manhiça, southern Mozambique, data from the ongoing surveillance of invasive bacterial infections showed that *S. aureus* is the leading cause of community-acquired neonatal bacteremia/sepsis, accounting for 39% (Sigaúque et al., 2009); however the molecular epidemiology and pathogenicity determinants remain unknown. Therefore, in the present study we aimed to determine genetic diversity and virulence factors of a collection of *S. aureus* causing community-acquired bacteremia in children less than 5 years admitted to Manhiça District Hospital, a rural area in southern Mozambique.

## Materials and methods

### Study area

The study was conducted by the Manhiça Health Research Centre (Centro de Investigação em Saúde de Manhiça—CISM) at Manhiça District Hospital, the referral health facility for Manhiça district in a rural area of Maputo province in southern Mozambique. Since 1996, CISM has been running a Demographic Surveillance System (DSS) for vital events and migrations in the population living within the study area covering approximately 95,000 inhabitants. In 2014, the study area was expanded to the whole District and currently covering 178,000 inhabitants. Each person living within the DSS study area is issued a unique Permanent Identification Number that describes the geographic location, household number and personal number within the household. A full description of the geographic and socio-demographic characteristics of the study community has been detailed elsewhere (Sacoor et al., 2013).

### Bacterial isolates

CISM has been carrying out high-quality surveillance of pediatric invasive bacterial infections since 2001 in jointly operation with the Manhiça District Hospital. For that, blood culture is routinely performed upon hospital admission in all children aged less than 2 years and for older children (up to 15 years) with axillary temperature ≥39°C, while culture of cerebrospinal fluid (CSF) is routinely performed for all children with suspected meningitis (Sigaúque et al., 2009).

For blood culture, 1–3 ml of whole blood from venous puncture were inoculated into a pediatric bottle (Pedibact®, Becton-Dickinson, Franklin Lakes, NJ, USA) and incubated into automatic system Bactec9050 (Becton-Dickinson, Franklin Lakes, NJ, USA), for 5 days. All positive cultures with a Gram stain compatible to *S. aureus* were sub-cultured into blood agar plates and incubated overnight at 37°C in a 5% CO_2_ atmosphere. Presumptive identification of Staphylococci was performed on the basis of colony morphology and β-hemolysis test. Colonies compatible with *S. aureus* were confirmed by catalase and third generation Pastorex coagulase (Hercules California, USA) test. Due to financial limitations associated to the cost of DNA microarray assay, we randomly selected 84 isolates (±20% of the total of positives) from 2001 to 2009 for molecular characterization and assessment of antimicrobial susceptibility.

### Species confirmation and antimicrobial susceptibility testing

Species identification was confirmed by MALDI-TOF mass spectrometry (BRUKER Daltonics GmbH, Bremen, Germany). Antimicrobial susceptibility was determined by VITEK 2 (bioMérieux, Marcy, France) for penicillin, oxacillin, cefuroxime, gentamicin, moxifloxacin, erythromycin, clindamycin, linezolid, daptomycin, vancomycin, rifampicin, and trimethoprim/sulfamethoxazole.

### Genetic characterization of *S. aureus* isolates

#### DNA microarray-based genotyping

A DNA microarray (Identibac® *S. aureus* Genotyping, Alere Tecnologies GmbH, Jena, Germany) containing 334 probes was used to detect diverse *S. aureus* pathogenicity markers, resistance determinants and virulence factors, following the manufacturer's instructions (http://www.alere-technologies.com). Briefly, genomic DNA was purified using the cell lysis components of the assay in combination with DNeasy blood and tissue kit (Qiagen, Hilden, Germany). The test is based on a linear multiplex primer elongation, using one primer for every single target and DNA labeling by incorporation of biotin-16-dUTP. Following DNA hybridization, microarray probes were washed, and then horseradish-peroxidase-streptavidin-precipitation reaction was performed resulting in visible gray spots in case of positive. The clonal complex (CC) of each isolate was automatically deduced from the hybridization gene patterns using the Array Mate reader (Iconoclust, Alere Technologies GmbH Jena, Germany) (Ruffing et al., 2012).

#### Spa typing

Sequencing of the hypervariable region of the *S. aureus* protein A gene (*spa*) was performed for all analyzed isolates using specific primers *spa*-1113F (5-TAAAGACGATCCTTCGGTGAGC-3) and *spa*-1618R (5-TTAGCATCTGCATGGTTTGC-3). For DNA extraction, the isolates were recovered from −70°C freezer, streaked on blood agar plates and incubated for 18–24 h in a 5% CO_2_ incubator. A loop full of bacterial colonies was suspended into 500 μl of distilled water and boiled for 10 min in a heat block and then centrifuged at 10,000 × g for 10 min. The supernatant was used as DNA template for PCR. *Spa* gene amplification was performed into 0.2 ml Eppendorf tubes in 25 μl reaction volume. Master Mix was prepared by adding 14.25 μl of bidistilled water, 5 μl (10X Dream Taq buffer), 2.5 μl (dNTP, 2.5 mM Roche), 0.25 μl of each primer (20 pmoles), 0.25 μl of Taq polymerase (Thermo Scientific Dream Taq DNA polymerase EP0702, USA); and 2.5 μl of DNA template. The amplification conditions were: first step at 95°C for 5 min, followed by 30 cycles of 95°C for 60 s, 60°C (60 s), 72°C (2 min), with a final extension at 72°C for 10 min and then 4°C hold. The amplified product was visualized in 2% agarose gel and digested with the Exo-SAP IT (Affymetrix, Cleveland, United States) for 37°C, 15 min and the reaction was stopped at 80°C for 15 min for DNA sequencing. *Spa* types sequences were analyzed using the Staph Type Ridom Software version 2.2.1 GmbH, Germany (Mellmann et al., 2007).

#### Data entry and statistical analysis

Clinical and epidemiological patients questionnaires were double entered in the FoxPro program (version 2.6, Microsoft Corporation, Redmond, Washington, USA) and discrepancies in data entries were resolved by referring to the original forms. DNA microarray data were entered exported to Excel file and converted for Stata and merged with the clinical and epidemiological data to create a master file. Mortality was calculated considering only those with known outcome excluding transferred or those that left hospital without medical consent. Statistical analyses were performed using STATA package software (version 14.1, STATA Corporation, College Station, Texas, USA). Proportions were compared using X^2^ test or Fischer Exact as appropriate.

## Results

### Clinical isolates and antimicrobial susceptibility

During the study period (January 2001 to December 2009), 32,488 blood cultures were performed from children less than 5 years yielding a positivity rate of 8% (*n* = 2,748) of which 398 (14%) of the positives were *S. aureus*. Eighty-four isolates (~20%) were randomly selected and characterized at molecular level. Age mean of the patients was 11 months (*SD* = 13.8). The majorities were neonates (25/84; 30%) and children from 1 to 11 months (24/84; 29%) followed by age group 12–23 (21/84; 27%) and 24–59 months (8/84; 10%). The isolates were highly resistant to the most commonly used antibiotics, with the highest rates being observed for penicillin (89%), followed by tetracycline (59%), trimethoprim sulfametoxazole (36%), clindamycin (21%), and erythromycin (21%). Resistance to oxacillin, cefuroxime, gentamycin and rifampicin accounted for 9% each antibiotic. There were no resistance observed for linezolid, daptomycin and vancomycin.

### Virulence profile for individual genes

An overview of the most relevant genes in the studied bacteremic *S. aureus* is provided in the Table S1. Table S2 shows the overall microarray raw data and antibiotic susceptibility for each individual isolates. The majority of the isolates possess the accessory gene regulator allele I (*agr*I) (45%) followed by *agr*II (28%), and *agr*III (17%). Two capsule types were detected (type 8 in 52% and 5 in 47% of the isolates). Antibiotic resistance genes were mostly of penicillin resistance (*blaZ*) with 91% followed by tetracycline (*tetK*) (49%) and macrolide/clindamycin (*ermC*) (20%). Other detected genes included mec*A* for methicillin resistance (MRSA) (8%), *aacA*-*aphD* (gentamycin/tobramycin), *dfrS*1 (trimethoprim) and *cat* (chloramphenicol), all with 9%, each.

Pyrogenic toxin super-antigen genes, were mostly of staphylococcal enterotoxin (SE) namely, enterotoxin G, enterotoxin-like gene/protein M (*selm*), N (*sen*), U (*selu*) all with 35%, each. The toxic shock syndrome toxin-1 (*tst1*) was detected in 16% of the isolates while the exfoliative toxins were mostly *etA* (14%) and *etD* (15%). The PVL encoding genes were detected in 30% of the analyzed isolates while α-hemolysin (98%), γ-hemolysin (97%) and δ-hemolysin were detected in all isolates (100%).

The transferrin-binding protein immune evasion precursor (*isdA*) was detected in all isolates (100%). Other precursor genes mostly found were of hyaluronatelyase (*hyaA* consensus) (98%), staphylococcal component inhibitor (*scn*) (97%), staphylokinase (*sak*) (80%) and chemotaxis-inhibiting protein (CHIPS) (63%). Additionally, predominant genes encoding for proteases were aureolysin (*aur*) (80%), serine proteases (A, B) (92%), glutatamylendopetidase (100%), and staphopain protease (100%).

Among biofilm precursor genes, the most important was *icaA* encoding for the intercellular adhesion protein A found in all isolates (100%) followed by the gene *icaD* encoding for the biofilm PIA synthesis protein D (98%). Genes encoding for adhesions proteins were highly diverse. The bone sialoprotein-binding protein (*bbp*), clumping factor A&B (*clfA*&*clfB*), cell surface elastin binding protein (*ebpS*), enolase (*eno*), fibronectin-binding protein A (*fnbA*) and van Willebrand factor binding protein (*vwb*) were detected in all analyzed isolates (100%).

### Genetic diversity

Thirty-eight different *spa* types and 14 CCs were identified among the analyzed isolates. *Spa* type t084 was the most prevalent with 10 isolates (11.9%) followed by t064 with 8 (9.5%), t002 and t1476 with 6 isolates each (7%), t186, t645, t701 each with 4.7% (4/84), t376, t3772 each with 3.6% (3/84), t015, t078, t127, t148, t2554, t2793, t5472, each with 2.4% (2/84) and 22 single *spa* types. Among CCs, CC8 was the most common with 21% followed by CC15 with 16% (Table 1). The association of CCs with antibiotic resistance mechanisms and virulence genes is summarized in Table 2. Noteworthy, MRSA isolates were exclusively grouped within CC8 while most of MSSA, resistance genes (*blaZ, tetk*), hemolysins (*hla, hld*), proteases (*splA, splB*) and adhesion precursors (*icaA, bbp, clfA, clfB*, and *fnbA*) were mostly common within CC8 or CC15. The accessory gene regulatory family was more diverse, being *agr*I more found in CC8 (47%) followed by CC25 (26%), *agr*II in CC15 (58%) and CC5 (37%). In contrast *agr*III was detected within CC1 (40%), CC88 (33%), and CC80 (26%) while *agr*IV was mostly detected in CC121 (71%). The toxic shock syndrome toxin 1 (*tst*1) was more common in CC8 (57%). PVL was more prevalent within CC5 (26%) followed by CC88 and CC121 each with 19%. The enterotoxin gene cluster (*egc*) was mostly found in CC25 (33%) and CC5 (30%) while *sea* was more common in CC8 (30%), CC5 and CC6 with 26% each. Among polysaccharide types, *cap5* was dominant for CC5 (45%) while *cap8* was more frequent in CC15 with 31% of the analyzed isolates. Exploratory analysis of the association of CCs with clinical outcome of patients showed that mortality tended to be higher among children infected by CC45 (33.3%; 1/3) and CC8 (27.8%; 5/18), although not statistical significant.

**Table 1:** **Clonal complexes (CC), *spa* types observed and outcome of the patients with *S. aureus* infection**.

**CC type**	**No. of strains (%)**	**No. different Spa types (%)**	**Spa types**	**Outcome**	
				**Died**	**Alive**
CC1	6/84 (7)	6/38 (15)	t10719, t127, t14473, t174, t1931, t8538	0	4/6 (66.6%)
CC12	1/84 (1)	1/38 (2)	t888	0	1/1 (100%)
CC121	5/84 (5)	3/38(7)	t14460, t2793, t645	0	4/5 (80%)
CC15	14/84 (16)	5/38 (13)	t064, t084, t11928, t1639, t774	1/14 (7.1%)	13/14 (92.8%)
CC152	1/84 (1)	1/38 (2)	t355	0	0
CC22	1/84 (1)	1/38 (2)	t891	0	0
CC25	10/84 (11)	6/38 (15)	t078, t14491, t2554, t258, t3772, t9045	1/10 (10%)	8/10 (80%)
CC45	3/84 (3)	2/38 (5)	t015, t2793	1/3 (33.3%)	2/3 (66.6%)
CC5	9/84 (10)	5/38 (13)	t002,t010, t045, t127, t645	0	9/9 (100%)
CC6	6/84 (7)	3/38 (6)	t2360, t304, t701	0	5/6 (83.3%)
CC8	18/84 (21)	5/38 (13)	t002, t064, t1476, t5472, t148	5/18 (27.8%)	8/18 (44.4%)
CC80	4/84 (4)	2/38 (5)	t376, t934	0	4/4 (100%)
CC88	5/84 (5)	2/38 (5)	t186, t690	0	2/5 (40%)
CC97	1/84 (1)	1/38 (2)	t426	0	0
TOTAL	84	38		8	60

**Table 2:** **Frequency of MRSA/MSSA and selected relevant genes according to clonal complexes within the analyzed bloodstream *S. aureus* isolates**.

**CC type**	**MRSA**	**MSSA**	**blaZ**	**tet (K)**	**agrI**	**agrII**	**agrIII**	**agrIV**	**tst1**	**PVL**	**sea**	**egc-cluster**	**hla**	**hld**	**sak**	**splA**	**splB**	**cap5**	**cap8**	**icaA**	**bbp, clfA, clfB, fnbA**
CC1	0 (0)	6 (7)	5 (6)	2 (4)	0 (0)	0 (0)	6 (40)	0 (0)	2 (14)	2 (7)	4 (17)	0 (0)	6 (7)	6 (7)	6 (8)	6 (7)	6 (7)	0 (0)	6 (13)	6 (7)	6 (7)
CC12	0 (0)	1 (1)	0 (0)	1 (1)	0 (0)	1 (4)	0 (0)	0 (0)	0 (0)	0 (0)	0 (0)	0 (0)	1 (1)	1 (1)	1 (1)	1 (1)	1 (1)	0 (0)	1 (2)	1 (1)	1 (1)
CC121	0 (0)	5 (6)	5 (6)	4 (6)	0 (0)	0 (0)	0 (0)	5 (71)	0 (0)	5 (19)	0 (0)	5 (16)	5 (6)	5 (5)	5 (7)	5 (6)	5 (6)	0 (0)	5 (11)	5 (5)	5 (5)
CC15	0 (0)	18 (18)	14 (18)	15 (25)	0 (0)	14 (58)	0 (0)	0 (0)	0 (0)	0 (0)	0 (0)	0 (0)	14 (16)	14 (16)	0 (0)	14 (17)	14 (17)	0 (0)	14 (31)	14 (16)	14 (16)
CC152	0 (0)	1 (1)	1 (1)	1 (1)	1 (2)	0 (0)	0 (0)	1 (14)	0 (0)	1 (3)	0 (0)	0 (0)	1 (1)	1 (1)	1 (1)	0 (0)	0 (0)	1 (2)	0 (0)	1 (1)	1 (1)
CC22	0 (0)	1 (1)	1 (1)	0 (0)	1 (2)	0 (0)	0 (0)	0 (0)	0 (0)	1 (3)	0 (0)	1 (3)	1 (1)	1 (1)	1 (1)	0 (0)	0 (0)	1 (2)	0 (0)	1 (1)	1 (1)
CC25	0 (0)	10 (12)	10 (13)	6 (10)	10 (26)	0 (0)	0 (0)	0 (0)	0 (0)	3 (11)	0 (0)	10 (33)	10 (11)	10 (11)	9 (13)	10 (12)	10 (12)	10 (25)	0 (0)	10 (11)	10 (11)
CC45	0 (0)	3 (3)	2 (2)	1 (1)	1 (2)	0 (0)	0 (0)	1 (14)	2 (14)	1 (3)	0 (0)	3 (10)	2 (2)	3 (3)	3 (4)	1 (1)	1 (1)	0 (0)	3 (6)	3 (3)	3 (3)
CC5	0 (0)	9 (11)	9 (11)	5 (8)	0 (0)	9 (37)	0 (0)	0 (0)	2 (14)	7 (26)	6 (26)	9 (30)	9 (10)	9 (10)	8 (11)	9 (11)	9 (11)	9 (22)	0 (0)	9 (10)	9 (10)
CC6	0 (0)	6 (7)	5 (6)	0 (0)	6 (15)	0 (0)	0 (0)	0 (0)	0 (0)	0 (0)	6 (26)	0 (0)	6 (7)	6 (7)	6 (8)	6 (7)	6 (7)	0 (0)	6 (13)	6 (7)	6 (7)
CC8	7 (100)	11 (14)	18 (23)	10 (16)	18 (47)	0 (0)	0 (0)	0 (0)	8 (57)	0 (0)	7 (30)	2 (6)	18 (21)	18 (21)	18 (26)	16 (20)	16 (20)	18 (45)	0 (0)	18 (21)	18 (21)
CC80	0 (0)	4 (5)	1 (1)	4 (6)	0 (0)	0 (0)	4 (26)	0 (0)	0 (0)	1 (3)	0 (0)	0 (0)	4 (4)	4 (4)	4 (5)	4 (5)	4 (5)	0 (0)	4 (9)	4 (4)	4 (4)
CC88	0 (0)	5 (6)	5 (6)	7 (11)	0 (0)	0 (0)	5 (33)	0 (0)	0 (0)	5 (19)	0 (0)	0 (0)	5 (6)	5 (5)	5 (7)	5 (6)	5 (6)	0 (0)	5 (11)	5 (5)	5 (5)
CC97	0 (0)	1 (1)	0 (0)	0 (0)	1 (2)	0 (0)	0 (0)	0 (0)	0 (0)	0 (0)	0 (0)	0 (0)	1 (1)	1 (1)	1 (1)	1 (1)	1 (1)	1 (2)	0 (0)	1 (1)	1 (1)
Total	7	77	76	41	38	24	15	7	14	26	23	30	83	84	68	78	78	40	44	84	84

## Discussion

This is one of the few studies in the sub-Saharan Africa and probable globally assessing the molecular profile of virulence markers of *S. aureus* causing bacteremia in children. Our data suggest the presence of high diversity of *S*. *aureus* causing bacteremia in Mozambican children, with a limited number of potential lethal clones particularly in young children. Although the sample size was small, the high case fatality rate among children infected with CC45 and CC8 compared to other CCs, may suggest possible differences on the degree of pathogenicity among CCs. The high prevalence of CC8 and CC15 in our study is consistent with findings from the African German multi-centric study conducted from 2010 to 2013 in Africa and Germany on infection biology and epidemiology of *S. aureus* (http://www.african-german-staph.net) (Ruffing et al., 2017). Similarly, these CCs have been consistently reported in patients with bacteremia in Germany (Rieg et al., 2013), Sweden (Rasmussen et al., 2013) and from different infection sites in Nigeria, Northern Africa (Shittu et al., 2012). Indeed, CC8 is among the major CCs which includes most of MRSA causing both nosocomial and hospital infections worldwide (Argudín et al., 2011). However, our isolates are from community-acquired infection as blood cultures were collected upon admission and no further blood culture was collected during the hospital stay of the patients; in addition none of the patients had previous hospitalization in the preceding 3 months.

Although PVL has been primarily associated with skin and soft tissue infections such as furunculosis and skin abscesses (Chiu et al., 2012), the prevalence found here is a matter of concern as we recently described a case of CA-MSSA necrotizing pneumonia complicated with multifocal osteomyelitis, pericardial effusion and endocarditis in a 6-year-old boy PVL positive hospitalized in our community with poor outcome. This prevalence (30%) is higher compared to 2.4% in developed countries (Aamot et al., 2012) and up to 25% in other African countries (Kechrid et al., 2011; Orth et al., 2013) among patients with community-acquired bacteremia. In fact, the distribution of PVL may vary from different geographical regions (Correa-Jiménez et al., 2016) and it has been observed that African *S. aureus* carries the highest rates of PVL compared to developed countries, although the reason for this scenario still is a matter of debate (Schaumburg et al., 2011). Additionally, all identified PVL encoding genes were among MSSA isolates although this toxin has been shown to be present in both MRSA and MSSA strains (Muttaiyah et al., 2010). The fact that all MRSA isolates belonged to the USA500 epidemic clone (data not shown) may explain the lack of PVL in our MRSA cohort, as USA500 strains also lacks the PVL encoding genes and other mobile genetic elements contributing to virulence and transmissibility (Roberts, 2014).

The high prevalence of pyrogenic toxin super-antigens support the hypothesis that classical pyrogenic toxin genes are common in *S. aureus* and as many as 73% of *S. aureus* carry at least one of the genes encoding a classical pyrogenic toxin (Shukla et al., 2010). The high proportion of these toxins was also reported in similar studies of *S. aureus* blood isolates from German (Becker et al., 2003; Rieg et al., 2013), Sweden (Rasmussen et al., 2013) and Gabon (Central Africa) (Schaumburg et al., 2011). In contrast, the exfoliative toxins, *etA* and *etD* were much more frequent in our study compared to others (0–8%) (Becker et al., 2003; Rasmussen et al., 2013; Rieg et al., 2013).

Most of the isolates possessed the accessory gene regulator I (*agr*I) which supports the observation that *agr* group I strains comprise a significant majority of clinical isolates (Sakoulas et al., 2002). In addition, as expected all isolates carried either capsule type 5 or 8 (Riordan and Lee, 2004; Fischer et al., 2014) being *cap8* the most frequent with some geographical variations (Fischer et al., 2014). The high prevalence of adhesion molecules in our strain collection may correlate with the fact that MSCRAMMs are crucial for establishment of *S. aureus* infections (Gordon and Lowy, 2008). Several MSCRAMMs shown to be important for the invasiveness are highly conserved into the staphylococcal genome (Rasmussen et al., 2013). The ClfA is the major fibrinogen binding protein of *S. aureus* and has been suggested to bind to the C-terminal region of fibrinogen γ-chain resulting in platelet aggregation or clumping of bacteria in plasma. The contribution of ClfA into *S. aureus* pathogenesis has been demonstrated in several animal models of infection including endocarditis, arthritis and sepsis while ClfB has been found to enhance the attachment of *S. aureus* to the anterior nares during colonization. Fibronectin-binding-proteins (FnBP) A and B enable *S. aureus* to adhere and invade a range of cell types including the epithelia, endothelia, fibroblasts and osteoblasts (Lacey et al., 2016).

Additionally, the high prevalence of proteases may help to explain the potential invasiveness of the studied isolates. Proteases are essential for cell invasion, destruction of host tissues and creation of metastasis to other sites (Gordon and Lowy, 2008). As expected, the hemolysin-α gene *(hla)* was present in almost all isolates, with some occasional exceptions probably due to mishybridization reactions while the gene encoding for δ-hemolysin was detected in all isolates. Similarly, *hlb* was also present at high frequency. Hemolysins are known to cause membrane damage of red blood cells (Wardenburg and Peptides, 2012).

Although *S. aureus* is recognized to be highly resistant to the most common used antibiotics, the rate of resistance to clindamycin in our study was very surprising. In contrast to erythromycin, which is commonly used, clindamycin is almost unavailable in the study area. Therefore, possible explanation for this scenario could be erythromycin-induced resistance. Indeed all clindamycin resistant isolates showed induction of phenotypic resistance which was confirmed by the presence of *ermC* gene coding for inducible resistance to macrolide-lincosamide-streptogramin antibiotics (Ii and Jorgensen, 2005; Prabhu et al., 2011). On the other hand, despite the fact that MRSA was detected in less than 10% of isolates, this finding is a matter of concern as MRSA are often associated with increased health care costs by either increasing the duration of patient hospitalization or need for often unavailable second line treatment (Rybak and LaPlante, 2005).

In summary, this is the first report of molecular characterization of *S. aureus* bacteremia in Mozambican children. These data provide a snapshot on the genetic diversity and pathogenicity markers of *S. aureus* causing pediatric bacteremia, which may explain its potential role as leading cause of neonatal bacteremia in our community. This emphasizes the urgent need of its recognition for prompt treatment. Further studies involving a large number of isolates are needed to explore in detail the potential impact of clonal complexes with patient outcome.

## Ethics statement

The strains characterized here were isolated from the ongoing invasive bacterial surveillance system which included several study protocols reviewed and approved by the Mozambican National Bioethics Committee for Health and institutional review boards of Hospital Clinic of Barcelona, Spain; the US Centers for Disease Control and Prevention and the University of Maryland School of Medicine.

## Author contributions

IM, BS, PA have been contributing in design of the study. MH, UR, LV have been contributing in experiment design of microarray. DV, MG, SA have been contributing in study implementation, seeing patients (SA) and laboratory experiments. All authors have contributing in writing and revision of the manuscript.

## Funding

This study has been supported by the grant of the Deutsche Forschungsgemeinschaft HE 1850/11-1 to MH and IM. CISM receives core funds from Spanish Agency for International Cooperation and Development (AECID).

### Conflict of interest statement

The authors declare that the research was conducted in the absence of any commercial or financial relationships that could be construed as a potential conflict of interest.
