# Supplementary material for: Molecular Characterization of Community Acquired Staphylococcus aureus Bacteremia in Young Children in Southern Mozambique, 2001–2009
Source: Front Microbiol. 2017 May 4;8:730. doi: 10.3389/fmicb.2017.00730 (PMC5415612; doi:10.3389/fmicb.2017.00730)
Supplement: Supplementary file 1 [file Table1.docx]

**Table S1.** DNA microarray genotype data for the most relevant genetic determinants for the 84 *S. aureus* analyzed isolates. Proportions were calculated using the number of positive isolates for each target gene diving by the total number of analyzed isolates.

| **Gene Category** | **Gene Description** | **Target or type** | **Proportions n (%)** |
| --- | --- | --- | --- |
| Resistance | alternate penicillin binding protein 2, defining MRSA | *mecA* | 7(8) |
|  | mercury resistance gene operon, Hg(II) reductase | *merA* | 6(7) |
|  | mercury resistance gene operon, alkyl-mercury lyase | *merB* | 7(8) |
|  | beta-lactamase | *blaZ* | 76(91) |
|  | beta lactamase repressor (inhibitor) | *blaI* | 76(91) |
|  | beta-lactamase regulatory protein | *blaR* | 76(91) |
|  | macrolide/clindamycin gene | *erm(C)* | 17(20) |
|  | gentamicin/tobramycin resistance gene | *aacA-aphD* | 8(9) |
|  | trimethoprim resistance gene | *dfrS1* | 8(9) |
|  | tetracycline resistance gene | *tet(K)* | 41(49) |
|  | tetracycline resistance gene | *tet(M)* | 9(10) |
|  | chloramphenicol resistance gene | *cat* | 8(9) |
|  | metallothiol transferase | *fosB* | 64(77) |
|  | quaternary ammonium compound resistance gene protein C | *qacC* | 3(3) |
|  | putative transport protein (=tetEfflux) | *sdrM* | 83(98) |
| Agr-type | accessory gene regulator allele I | *agrI* | 38(45) |
|  | accessory gene regulator allele II | *agrII* | 24(28) |
|  | accessory gene regulator allele III | *agrIII* | 15(17) |
|  | accessory gene regulator allele IV | *agrIV* | 7(8) |
| Toxin genes | toxic shock syndrome toxin 1 | *tst1* | 14(16) |
|  | enterotoxin A (=entA) | *sea* | 23(27) |
|  | enterotoxin B (=entB) | *seb* | 14(16) |
|  | enterotoxin C (=entC) | *sec* | 14(16) |
|  | enterotoxin G (=entG) | *seg* | 30(35) |
|  | enterotoxin H (=entH) | *seh* | 6(7) |
|  | enterotoxin I (=entI) | *sei* | 25(29) |
|  | enterotoxin J (=entJ) | *sej* | 8(9) |
|  | enterotoxin K (=entK) | *sek* | 12(14) |
|  | enterotoxin L (=entL) | *sel* | 13(15) |
|  | enterotoxin-like gene/protein M (=sem, entM) | *selm* | 30(35) |
|  | enterotoxin-like gene/protein N (=sen, entN), consensus probe | *seln* | 30(35) |
|  | enterotoxin-like gene/protein O (=seo, entO) | *selo* | 28(33) |
|  | enterotoxin gene cluster | *egc* | 30(35) |
|  | enterotoxin Q (=entQ) | *seq* | 12(14) |
|  | enterotoxin R (=entR) | *ser* | 7(8) |
|  | enterotoxin-like gene/protein U (=seu, entU) | *selu* | 30(35) |
|  | enterotoxin-like protein ORF CM14 | *ORF CM14_ probe1* | 7(8) |
|  | exfoliative toxin serotype A | *etA* | 12(14) |
|  | exfoliative toxin D | *etD* | 13(15) |
| Leukocidin | Panton Valentin Leukocidin | *PVL* | 26(30) |
| Hemolysin | hemolysin gamma, component A | *hlgA* | 82(97) |
|  | putative membrane protein | *hl* | 82(97) |
|  | hemolysin alpha | *hla* | 83(98) |
|  | putative membrane protein  hemolysin delta | *hlIII*  *hld* | 83(98)  84(100%) |
|  | hemolysin beta | *hlb_probe 1* | 63(75) |
|  | hemolysin beta | *hlb_probe 2* | 67(79) |
|  | hemolysin beta | *hlb_probe 3* | 66(78) |
|  | hemolysin beta without phage insertion | *un-disrupted hlb* | 3(3) |
| Immune evasion | staphylokinase | *sak* | 68(80) |
|  | chemotaxis-inhibiting protein (CHIPS) | *chp* | 53(63) |
|  | staphylococcal complement inhibitor | *scn* | 82(97) |
|  | epidermal cell differentiation inhibitor | *edinA* | 7(8) |
|  | epidermal cell differentiation inhibitor B | *edinB* | 15(17) |
|  | immunodominant antigen B | *isaB* | 78(92) |
|  | defensin resistance gene protein | *mprF (COL+MW2)* | 59(70) |
|  | transferrin-binding protein | *isdA* | 84(100) |
|  | hypothetical protein, similar to integral membrane protein LmrP | *lmrP (other than RF122)_probe1* | 77(91) |
|  | type I site-specific desoxyribonuclease subunit, 2nd locus | *hsdS2 (Mu50+N315+COL+USA300+NCTC8325)* | 33(39) |
|  | type I site-specific desoxyribonuclease subunit, 3rd locus | *hsdS3 (all other than RF122+ MRSA252)* | 55(65) |
|  | type I site-specific desoxyribonuclease subunit, unknown locus | *hsdSx (CC25)* | 62(73) |
|  | hypothetical protein, located next to serine protease operon | *Q2FXC0* | 36(42) |
|  | Unspecific efflux/transporter | *Q2YUB3* | 20(23) |
|  | hypothetical protein | *Q7A4X2* | 30(35) |
| Proteases | aureolysin | *aur* | 68(80) |
|  | serinprotease A | *splA* | 78(92) |
|  | serinprotease B | *splB* | 78(92) |
|  | serinprotease E | *splE* | 51(60) |
|  | glutamylendopeptidase | *sspA* | 84(100) |
|  | staphopain B, protease | *sspB* | 84(100) |
|  | staphopain A (staphylopain A), protease | *sspP* | 84(100) |
| Capsule type | Capsule type 5 | *cap5* | 40(47) |
|  | Capsule type 8 | *cap8* | 44(52) |
| Biofilm | intercellular adhesion protein A | *icaA* | 84(100) |
|  | intercellular adhesion protein C | *icaC* | 83(98) |
|  | biofilm PIA synthesis protein D | *icaD* | 83(98) |
| Adhesion | bone sialoprotein-binding protein | *bbp* | 84(100) |
|  | clumping factor A | *clfA* | 84(100) |
|  | clumping factor B | *clfB* | 84(100) |
|  | collagen-binding adhesin | *cna* | 23(27) |
|  | cell wall associated fibronectin-binding protein | *ebh* | 83(98) |
|  | cell surface elastin binding protein | *ebpS* | 84(100) |
|  | enolase | *eno* | 84(100) |
|  | fibronectin-binding protein A | *fnbA* | 84(100) |
|  | fibronectin-binding protein B | *fnbB* | 82(97) |
|  | major histocompatibility complex class II analog protein (=Extracellular adherence protein, eap) | *map* | 81(96) |
|  | Staphylococcus aureus surface protein G | *sasG* | 65(77) |
|  | Ser-Asp rich fibrinogen-/bone sialoprotein-binding protein C | *sdrC* | 83(98) |
|  | Ser-Asp rich fibrinogen-/bone sialoprotein-binding protein D | *sdrD* | 76(90) |
|  | van Willebrand factor binding protein | *vwb* | 84(100) |
